# Analysis of the Cytotoxic Potential of Anisomelic Acid Isolated from *Anisomeles malabarica*

**DOI:** 10.3797/scipharm.1210-15

**Published:** 2013-01-25

**Authors:** Christo Paul Preethy, Ali Abdullah Alshatwi, Muthukumaran Gunasekaran, Mohammad Abdulkadher Akbarsha

**Affiliations:** 1 Department of Animal Science, Bharathidasan University, Tiruchirappalli 620024, India.; 2 Department of Food Science and Nutrition, King Saud University, Riyadh 11451, Saudi Arabia.; 3 Department of Biology, Fisk University, Nashville, TN 37208, USA.; 4 Mahatma Gandhi-Doerenkamp Center, Bharathidasan University, Tiruchirappalli 620024, India.

**Keywords:** *Anisomeles malabarica*, Anisomelic acid, Apoptosis, Cytotoxicity, Anti-cancer

## Abstract

Anisomelic acid (AA), one of the major compounds in *Anisomeles malabarica*, was tested for its cytotoxicity and apoptosis-inducing potential in breast and cervical cancer cells. The MTT assay for cell viability indicated that AA is cytotoxic to all of the four cell lines tested in a dose- and duration-dependent manner. Acridine Orange & Ethidium Bromide (AO & EB) and Hoechst 33258 staining of AA-treated cells revealed typical apoptotic morphology such as condensed chromatin and formation of apoptotic bodies. The comet assay revealed DNA strand break(s), indicating that AA induces DNA damage which culminates in apoptosis. Thus, the study revealed the anti-proliferative and apoptosis-inducing properties of AA in both breast and cervical cancer cells. Therefore, anisomelic acid offers potential for application in breast and cervical cancer therapy.

## Introduction

Throughout history, natural products have offered a rich source of compounds that have found many applications in the fields of medicine, pharmacy, and biology. With special reference to cancer, several promising anti-cancer molecules have been discovered through screening natural products from plants, animals, marine organisms, and microorganisms. Since an ensured therapy for cancer is yet to emerge, interest in finding newer anti-cancer molecules among natural products is sustained. Vincristine, irinotecan, etoposide, and paclitaxel are examples of plant-derived anticancer compounds [1]. Plants contain different types of secondary metabolites, out of which terpenoids are involved in a multitude of ecological and physiological functions. Their major function in plants is chemical defense against insects and environmental stress, but they are also involved in the repair of wounds and injuries. Plant-derived terpenoids possess antioxidative activities, but they also possess a variety of specific properties since they undergo distinct interactions with several regulatory proteins. Taxol is a complex polyoxygenated diterpenoid which has been used clinically to combat several cancer diseases with the generic name of paclitaxel with excellent activity against breast and ovarian cancers [2].

Anisomelic acid, one of the major compounds in *Anisomeles malabarica* (L.) R. Br., is a cembrane-type diterpenoid, which can be synthesized chemically [3]. Anisomelic acid has also been previously isolated from *A. malabarica*[4] and *A. indica* and it has been shown to be cytotoxic to KB cells [5, 6]. *A. malabarica* has been shown to possess many other compounds viz., Anisomelolide, Malabaric Acid, 2-Acetoxymalabaric Acid, Anisomelyl Acetate, Anisomelol, etc. [7, 8]. However, to date, anisomelic acid (AA) has not been tested against estrogen receptor (ER)-negative and -positive breast cancer cells or HPV-positive cervical cancer cells, the types of cancers that are still challenging in terms of treatment. Therefore, developing upon the ethno-medical and scientific information so far available, the present study was undertaken to evaluate the cytotoxic property of AA in the breast and cervical cancer cell lines.

## Results and Discussion

**Fig. 1:**
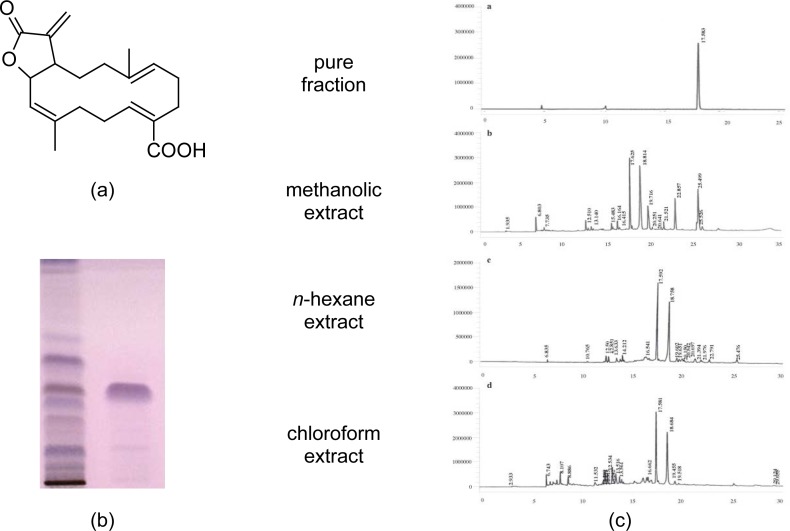
(a) Structure of anisomelic acid. (b) HPTLC chromatogram showing the bands of the crude methanolic extract of *A. malabarica* and AA. (c) HPLC chromatogram of AA, and crude methanolic, *n*-hexane and chloroform extracts of A. malabarica. AA shows as a single peak with retention time of 17.5 min, and the peak is found in all the three crude extracts.

AA was isolated as a colorless, crystalline compound, and was confirmed based on existing literature [4] adopting UV, FT-IR, and ^1^H NMR, ^13^C. Its molecular formula is C20H26O4 and molecular weight 330 (Fig 1a). The compound appeared as a single band on HPTCL (Fig 1b) and as a single peak on RPHPLC (Fig 1c). The presence of the compound in the methanol, *n*-hexane, and chloroform extracts was also confirmed by RPHPLC where the compound showed a retention time of 17.5 min (Fig. 1c).

**Fig. 2:**
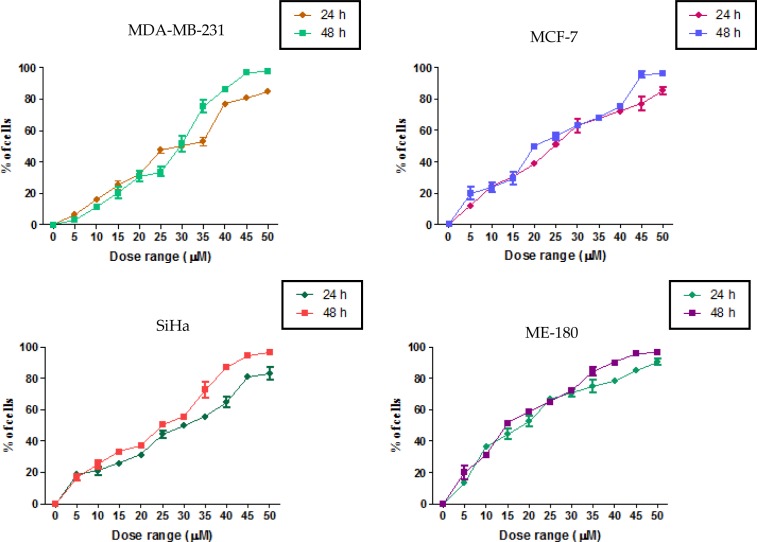
Cytotoxicity of AA on the four cell lines. Cytotoxicity was determined by the MTT assay and calculated as a percentage of inhibition on cell proliferation.

The MTT assay was conducted as an indirect measure to determine the viability of cells treated with AA. Anisomelic acid was found to be cytotoxic to the cells. The IC_50_ of the compound for all of the four cell lines is given in the Table 1. As shown in Figure 2, AA affected the viability of cells in a dose- and duration-dependent manner. The efficacy of AA against the four cell lines tested was in the order ME>MCF7>SiHa>MDA-MB-231. AA did not elicit apoptosis in non-cancerous cells (Data presented elsewhere).

**Fig. 3:**
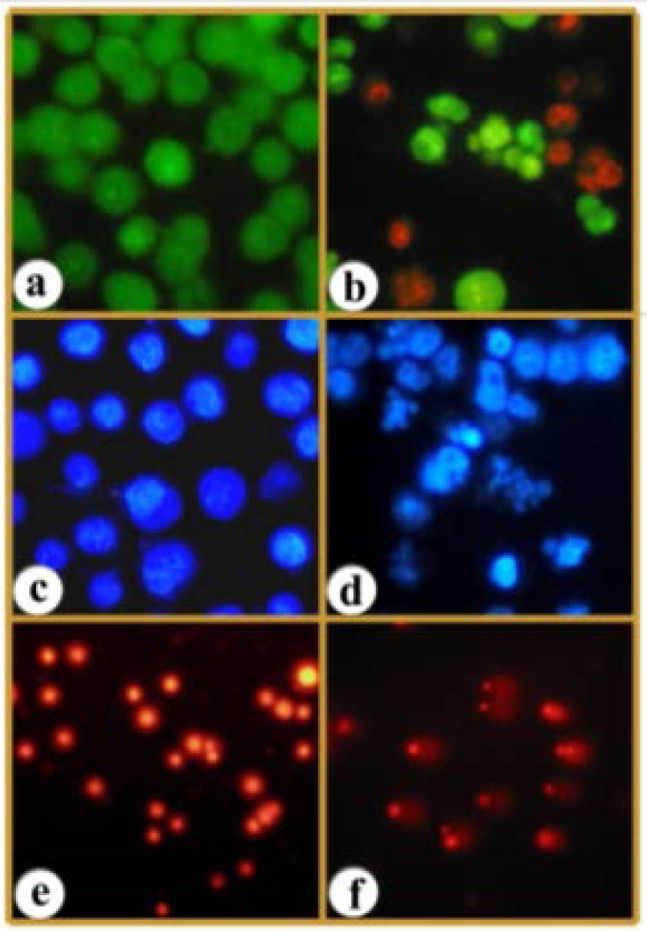
Photomicrographs showing untreated MDA-MB-231cells (a, c, e) and AA (48 h) treated cells (b, d, f) stained with AO & EB (a, b), Hoechst 33258 (c, d) and tested by the comet assay and stained with EB (e, f).

AO & EB and Hoechst staining were adopted to infer if the cells responded to the treatment of AA with apoptosis and/or necrosis. Staining of cells with AO & EB indicated typical apoptotic morphology such as chromatin condensation, nuclear fragmentation, and apoptotic body formation (Fig. 3 a, b). Hoechst 33258 staining revealed shrinkage of cells, fragmentation of nuclei, and formation of apoptotic bodies (Fig. 3 c, d).

We adopted the single cell gel electrophoresis technique that detects DNA damage in individual cells [10]. The control cells showed little, if any, comets. But the treatments produced a high percentage of damaged cells forming comets (Fig. 3 e, f). From one perspective, DNA strand breaks as revealed by the comet assay is taken to indicate genotoxicity. However, the from cancer perspective, the outcome of the testing should be loss of viability, cell cycle arrest and/or death of the cancer cells. DNA damage, if not repaired, will lead to cell cycle arrest and cell death, which is invariably apoptosis. Thus, DNA damage and apoptosis are closely linked [9]. Our aim in conducting the comet assay was essentially to assess DNA damage, as induced by AA, and the corollary viz., apoptosis.

Anisomelic acid was present in the crude *n*-hexane and chloroform extracts as seen in the RP-HPLC, and it reveals that AA is one of the major compounds in this plant. Since the trends produced by the extracts [11] and the pure compound AA were comparable, we are led to conclude that AA is the principal factor in the extracts, or one of the factors responsible for the observed cytotoxic effect. AA is a terpenoid, and a broad range of the biological properties of terpenoids is described, including cancer chemopreventive effects, antimicrobial, antifungal, and antiviral [2]. This study shows that AA is cytotoxic to the cancer cells. There are many studies showing that terpenoids elicit significant bioactivity in the similar concentration range or higher than the range of anisomelic acid we tested (10– 50 μM or 3.25–16.25 μg/mL). For example, Geraniol (50–400 μmol/L), Andrographolide (3–100 μmol/L), Excisanin A (1–32 μmol), and Escin (10–60 μg/mL) showed bioactivity *in vitro* at concentrations closer to or higher than in our study [12]. The above compounds have also been tested for *in vivo* activity, deriving the dose from the *in vitro* assays, and have been found to exert both antiproliferative and cytotoxic effects. In fact, there is always the approach of *in vitro* to *in vivo* extrapolation [13]. Thus, the effective *in vivo* dose of AA, to be derived from *in vitro* data, will be appropriate and practicable.

Thus, in conclusion, we could demonstrate that anisomelic acid of *A. malabarica* affects the viability of and induces cell death in ER-positive and –negative breast cancer cells and HPV16-positive cervical cancer cells, irrespective of the variable genetic property underlying the malignancy, but the extent of response appears to have a bearing on the genetic constitution of the cells. The molecular mechanism behind the action of AA is currently under investigation.

## Experimental

### Isolation of the active compound

AA was isolated from *A. malabarica* according to the protocol by Purushothamam *et al.*, [4], with the slight modification of adopting Medium Pressure Liquid Chromatography (MPLC). The shade-dried plants were powdered and 2 kg powder was extracted thrice in MeOH with intermittent stirring, and the extract was pooled and evaporated under reduced pressure in a rotary evaporator. The yield of the MeOH extract was 120g. The methanolic extract was partitioned between *n*-hexane and MeOH (1:1) to give *n*-hexane extract (8g) and MeOH (85g) extract. The partitioned MeOH extract was chromatographed on silica gel (230–400 mesh) and eluted on Medium Pressure-Liquid Chromatography (MPLC) with 100% *n*-hexane to yield a mixture of oil fractions. This was followed by successive elutions with a gradient of *n*-hexane, CHCl3, and MeOH which resulted in 140 fractions. Each fraction was spotted on a pre-coated silica gel (Merk-60 F254, 0.25mm thick) and eluted in *n*-hexane : ethyl acetate : MeOH (8:1.5:0.5), sprayed with *p*-anisaldehyde, and observed under UV light. Fractions with similar color and Rf values in HPTLC were pooled and this culminated in six fractions, and fraction 4 yielded a single major band. This fraction was recrystallized from methanol and a colourless crystalline compound was obtained.

### Chromatographic analysis of AA and identification of the compound

The crude methanolic, *n*-hexane, and chloroform extracts were subjected to RP-HPLC, and the purity and presence of the isolated compound in the extracts were confirmed by HPTLC and RP-HPLC. The RP-HPLC was performed on a Shimadzu HPLC system equipped with a reverse phase C18 column and a photodiode array detector. The separation was carried out on a Phenomenox column (5μ, 4.6 x 250 mm) using water (solvent A) and acetonitrile (solvent B) as solvents. Gradient elution started with 33.7% solvent B for 0.01 min, was changed to 33.7% solvent B for 5 min, then to 50% solvent B for 8 min, then to 70% solvent B for 12 min, and finally changed to 100% solvent B for 20 min. The flow rate was 1.0 ml/min and the detection was done at 220 nm. The compound was identified using UV, FT-IR, CHNO elemental analysis, ^1^H and ^13^C NMR, and by comparison of the spectral data with that reported in the literature.

### Determination of the cytotoxic property of AA by MTT assay

To evaluate the cytotoxic property of anisomelic acid, the MTT colorimetric assay was performed. AA was dissolved in DMSO (Sigma Chemical Co., St. Louis, MO, USA). The cells were seeded in 96-well plates at a density of 5 × 10^4^ cells/well and treated with AA at the concentration range 0–50 μM, at 10 μM interval, at 37°C, for 24 and 48 h. Cisplatin (Getwell Pharmaceuticals, India), was included in the assay as a reference anticancer agent. At the end of the exposure period, the cells were subjected to assessment of viability by adopting the MTT assay. The percentage inhibition was calculated from this data, using the formula:

$$\frac{\text{Mean OD of untreated cells}\left(\text{control}\right)-\text{Mean OD untreated cells}}{\text{Mean OD of untreated cells}\left(\text{control}\right)}$$

The IC_50_ concentration was determined as the dose that would be required to kill 50% of the cells with the respective preparation and duration.

### Fluorescent staining for morphological assessment of cells

For further studies, only the cell line with the highest IC_50_ concentration, MDA-MB-231, was selected to assess the mode of cell death induced by AA. MDA-MB-231 cells were seeded in 12-well plates and allowed to reach 70% confluence. The cells were then treated with the IC_50_ concentration of AA (which produced the best results as revealed in the MTT assay) and incubated for 48 h. After incubation, the cells were stained with AO & EB and Hoechst 33258 as given in 3.3.3 and analyzed in a fluorescent microscope (Carl Zeiss, Jena, Germany).

### Comet assay

DNA damage was quantified adopting the comet assay and CASP software. The images were used to estimate the DNA content of individual nuclei and to evaluate the degree of DNA damage representing the fraction of total DNA in the tail. The cells were assigned to five classes: 0 (<7% of the DNA in the tail damaged), 1 (7–15%), 2 (15–22%), 3 (22–30%), and 4 (>30%, maximally damaged).

## Figures and Tables

**Tab. 1.** IC_50_ values of anisomelic acid on cancer cell lines

	**MDA-MB-231 (μM)**	**MCF-7 (μM)**	**SiHa (μM)**	**ME-180 (μM)**
24 h	43.56 ± 2.4	27.56 ± 1.4	33.4 ± 3.6	22.23 ± 4.3
48 h	41.23 ± 4.4	25.34 ± 2.3	29.56 ± 2.1	18.34 ± 2.6
